# Addition of preoperative transversus abdominis plane block to multimodal analgesia in open gynecological surgery: a randomized controlled trial

**DOI:** 10.1186/s12871-023-01981-w

**Published:** 2023-01-12

**Authors:** Zhi Yu Geng, Yan Zhang, Hui Bi, Dai Zhang, Zheng Li, Lu Jiang, Lin Lin Song, Xue Ying Li

**Affiliations:** 1 Department of Anesthesiology, Peking University First Hospital, Beijing, China; 2 Department of Obstetrics and Gynecology, Peking University First Hospital, Beijing, China; 3 Department of Biostatics, Peking University First Hospital, Beijing, China

**Keywords:** Gynecologic, Multimodal analgesia, Postoperative analgesia, Recovery Transversus abdominis plane block

## Abstract

**Background:**

Transversus abdominis plane (TAP) block can provide effective analgesia for abdominal surgery. However, it was questionable whether TAP had additional effect in the context of multimodal analgesia (MMA). Therefore, this study aimed to assess the additional analgesic effect of preoperative TAP block when added to MMA protocol in open gynecological surgery.

**Methods:**

In this prospective, randomized-controlled trial, 64 patients scheduled for open gynecological surgery were randomized to receive preoperative TAP block (Study group, *n* = 32) or placebo (Control group, *n* = 32) in addition to MMA protocol comprising dexamethasone, acetaminophen, flurbiprofen and celecoxib, and rescued morphine analgesia. The primary outcome was rescued morphine within 24 h after surgery. Secondary outcomes included pain scores, adverse effects, quality of recovery measured by 40-item quality of recovery questionnaire score (QoR-40) at 24 h, and quality of life measured with short-form health survey (SF − 36) on postoperative day (POD) 30.

**Results:**

The Study group had less rescued morphine than the control group within 24 h [5 (2–9) vs. 8.5 (5–12.8) mg, *P* = 0.013]. The Study group had lower pain scores at 1 h [3 (2–4) vs. 4 (3–5), *P* = 0.007], 2 h [3 (2–4) vs. 3.5 (3–5), *P* = 0.010] and 6 h [3 (2–3) vs. 3 (2.3–4), *P* = 0.028], lower incidence of nausea at 48 h (25.8% vs. 50%, *P* = 0.039), and higher satisfaction score [10 (10–10) vs. 10 (8–10), *P* = 0.041]. The SF-36 bodily pain score on POD 30 was higher in the Study group (59 ± 13 vs. 49 ± 16, *P* = 0.023).

**Conclusions:**

Preoperative TAP block had additional analgesic effect for open gynecological surgery when used as part of multimodal analgesia. Rescued morphine within 24 h was significantly reduced and the SF-36 bodily pain dimension at 30 days after surgery was significantly improved.

**Trial registration:**

www.chictr.org.cn (ChiCTR2000040343, on Nov 28 2020).

## Background

Enhanced recovery after surgery (ERAS) protocols are designed to minimize the stress response created by surgery and to improve patient recovery after surgery [1]. Avoiding opioid use with a multimodal pain management is an essential component of most ERAS pathways. A standardized multimodal analgesia (MMA) protocol with nonopioid agents or techniques could decrease opioid-related adverse effects such as nausea and vomiting, thus improve functional recovery after surgery [2, 3].

Ultrasound-guided transversus abdominis plane (TAP) block is performed by injecting local anesthetic between the muscle layers of the trunk. As an effective pain relief technique, it has been performed in various abdominal surgical procedures. Current evidence has revealed consistent results in terms of reducing pain and opioid requirements after surgery, especially for gynecologic surgery, appendectomy, and bariatric surgery [4–6]. However, in the context of MMA, the role of TAP block showed variable results in different procedures [7–9].

The analgesic efficacy of the TAP block is also influenced by several factors such as dose of local anesthetics, different approach used, and inclusion of co-analgesics [10, 11]. Whether performing the TAP block before or after the surgery has an important impact on analgesic control [12]. The posterior TAP block technique resulted in greater reduction in opioid requirement than the lateral approach [13].

Open gynecological surgeries are known to cause moderate to severe postoperative pain. In this randomized controlled study, we aimed to investigate whether adding preoperative TAP block to MMA protocol would confer additional benefit after open gynecologic surgery for benign indications. We hypothesized that preoperative TAP block had further preventive analgesic effect when used as part of MMA. The primary outcome was rescued morphine within 24 h after surgery. The secondary outcomes were pain scores, adverse effects and quality of recovery measured by a 40-item quality of recovery score (QoR-40) at 24 h and the short-form health survey (SF-36) on postoperative day 30.

## Methods

### Study design

This randomized clinical trial was performed at a tertiary university hospital in China. The study was approved by the Ethics committee of Peking University First Hospital (Reference Number: 2020–247, on 30/10/2020) and registered prior to patient enrollment at Chinese Clinical Trial Registry (ChiCTR2000040343, on 28/11/2020). Written informed consent was obtained from all patients before enrollment. The principles of Declaration of Helsinki were followed for this study. This manuscript adheres to the applicable CONSORT guidelines.

### Study population

From November 2020 to August 2021 female patients undergoing elective open gynecological surgery were screened for inclusion in the study. Eligible subjects were patients aged between 18 and 65 years and American Society of Anesthesiologists (ASA) physical status I-II. Exclusion criteria were: significant cardiovascular, gastric ulcer, hepatic, or renal disease; preoperative treatment with opioids or corticosteroids; allergies or contraindications to any drug used in the study, pregnancy or breastfeeding, or refusal to participate in the study.

### Randomization and blinding

After written informed consent was obtained, participants were randomly assigned to the study group or the control group. Randomization was carried out using a computer-generated random number list with a 1:1 ratio. Group assignment was performed using opaque, sealed envelopes prepared by a research nurse. On the day of surgery, before entering the operating room, the nurse (GYG) not involved in patient recruitment or data collection opened the envelope and prepared the study solutions. The patients, anesthesiologists and research assistant recording postoperative data were blinded to the group allocation throughout the study period.

### Standard general anesthesia management

All patients received a standardized general anesthetic technique. Anesthesia was induced with IV midazolam 0.03 mg/kg, propofol 1.5–2 mg/kg and sufentanil 0.2 μg/kg. Rocuronium 0.6 mg/kg was given to facilitate supreme laryngeal mask insertion. Maintenance of anesthesia was performed with continuous infusion of propofol, target-controlled infusion of remifentanil 2–3 ng/ml, and an intermittent supplemental sufentanil bolus of 0.1 μg/kg, titrated to keep mean blood pressure within 20% of the baseline values, and bispectral index (BIS) values between 40 and 60. Positive pressure ventilation was controlled to maintain an end-tidal carbon dioxide partial pressure between 35 and 55 mmHg. All patients received tropisetron 5 mg IV 30 min before the end of surgery for postoperative nausea and vomiting prophylaxis. At the end of surgery, muscle relaxation was reversed with neostigmine and atropine. Patients were extubated and transferred to the post-anesthesia care unit (PACU) for observation.

### Study protocol

After induction of anesthesia and before surgery, an ultrasound-guided bilateral posterior TAP block was performed by a single, experienced anesthesiologist. Patients were randomly allocated for bilateral injection with one of the following solutions: 20 mL ropivacaine 0.375% (Study group) or the same volume of 0.9% saline (Control group). The block was performed under ultrasonography guidance using a linear probe (6–13 MHz, GE) and a 22-gauge 0.71 × 80 mm needle (Stimuplex D, B-Braun Melsungen AG, Germany) after sterilizing the skin. Following verification of the position of the needle tip, the study solution was injected between the internal oblique and transversus abdominis plane under real-time imaging. The anesthesiologist who performed the block was not involved in patient allocation and assessment during the study period.

Both groups received MMA included dexamethasone, acetaminophen, flurbiprofen and celecoxib. All patients received dexamethasone 5 mg and flurbiprofen 50 mg IV immediately before skin incision as preemptive analgesia. After admission to the PACU, pain intensity was assessed with an 11- point numerical rating scale (NRS) (0 meant no pain, and 10 was the worst pain imaginable) by an investigator who was blinded to group allocation. IV boluses of morphine 1–2 were given as a rescue opioid for NRS pain score ≥ 4. Nausea or vomiting was treated with 5 mg IV metoclopramide. Rescue antiemetics were administered on the following conditions: two or more episodes of vomiting or retching, any nausea lasting for more than 30 min, a ‘severe’ degree of nausea or whenever treatment was requested by the patient.

After discharge from the PACU, a morphine intravenous patient-controlled analgesia (PCA) was provided as rescue analgesia (no background infusion, 1 mg bolus with a 6-min lockout interval). Another flurbiprofen 50 mg IV was administered 6 h after surgery. All patients received regular oral acetaminophen (650 mg every 8 h), and oral celecoxib (200 mg every 12 h) on postoperative day (POD) 1 and 2.

All data were collected by an investigator who was blinded to the group assignment and not involved in patient’s perioperative care. The observation period started from the end of surgery. The researcher assessed the patients at 1, 2, 6, 24 and 48 h after surgery. After discharge, research personnel contacted patients via we-chat on POD 30 to complete the SF-36 Health Survey Questionnaire.

### Outcome measures

The primary outcome was rescued morphine within 24 h after surgery. The secondary outcomes included: (1) NRS scores; (2) morphine consumption at other time points; (3) time to first ambulation and flatus；(4) adverse effects, such as nausea, vomiting, dizziness, and pruritus within 48 h after surgery; (5) quality of recovery assessed with QoR-40 score at 24 h after surgery; (6) patient satisfaction score (0 = totally unsatisfied, 10 = total satisfied) at 48 h; (7) quality of life measured with SF-36 health survey questionnaire on POD 30.

### Sample size calculation

Sample size calculation was based on previous studies involving the use of TAP blocks in patients undergoing open abdominal surgery [14, 15]. The average 24 h morphine requirement was approximately 20 mg. Assuming a common standard deviation of 8 mg, we estimated that a sample size of 29 patients per group would provide 80% power to detect a 30% difference in 24 h morphine consumption between the two groups at an α level of 0.05. To allow for a possible 10% dropout rate, a total of 64 patients (32 patients per group) were recruited.

### Statistical analysis

The Shapiro-Wilk test was used to test the hypothesis of normal distribution. Normally distributed continuous variables were expressed as the means (standard deviation), and analyzed with a student’s two-sample *t* test. Nonnormally distributed variables were expressed as medians (interquartile ranges [IQRs]), and analyzed using the Mann-Whitney *U* test. Categorical variables were described as numbers (percentages) and analyzed using the chi- square test or Fisher’s exact test as appropriate. Statistical analysis was performed using IBM SPSS version 22.0 (IBM). All tests were two-sided and *P* values less than 0.05 were considered statistically significant.

## Results

**Fig. 1:**
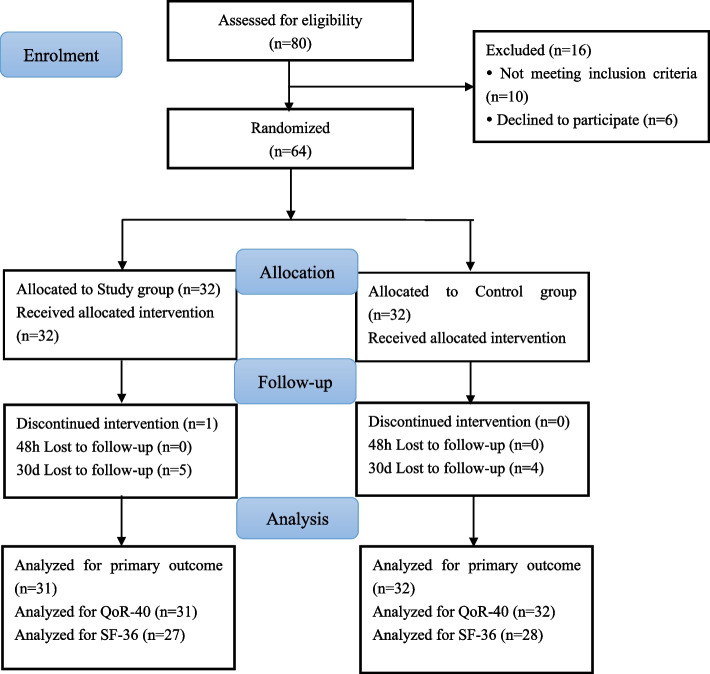
Participant flowchart

A total of 80 patients were assessed for eligibility, and 64 patients were enrolled and randomized to one of the two groups. One patient in the Study group was excluded due to discontinued intervention, thus 63 patients finally completed the study. Of the 64 patients included in the analysis, 36 had total abdominal hysterectomy and bilateral salpingectomy and 28 underwent myomectomy. The CONSORT flow diagram is presented in Fig. 1. Patient demographics and surgical data did not differ between the two groups (Table 1).

**Table 1:** Patient characteristics and surgical data

Variables	Study Group (*n* = 32)	Control Group (*n* = 32)	*P* value
Age (year)	44.5 ± 6.4	44.9 ± 8.0	0.837
Height (cm)	163.7 ± 5.8	162.7 ± 5.2	0.499
Weight (kg)	65.3 ± 11.7	64.8 ± 9.7	0.844
BMI (kg/m^2^)	24.3 ± 3.8	24.5 ± 3.5	0.896
ASA physical status I/II (n)	8/24	12/20	0.281
PONV score	3.4 ± 0.6	3.5 ± 0.5	0.244
Type of surgery(n/%)			0.614
TAH	17 (53.1%)	19 (59.4%)	
Myomectomy	15 (46.9%)	13 (40.6%)	
Type of incision (n/%)			0.777
Transverse incision	24 (75.0%)	23 (71.9%)	
Vertical incision	8 (25.0%)	9 (28.1%)	
Anesthesia time (min)	124.0 (103.3–141.5)	111.5 (101–141.5)	0.405
Operation time (min)	100.0 (73.5–113.0)	90.5 (81.5–103.0)	0.930
Intraoperative remifentanil (μg·kg^− 1^·h^− 1^)	6.1 ± 1.3	5.9 ± 0.8	0.531
Intraoperative sufentanil (μg/kg)	0.3 ± 0.1	0.3 ± 0.1	0.415

*Abbreviations*: *ASA* American Society of Anesthesiologists, *BMI* body mass index, *TAH* total abdominal hysterectomy and bilateral salpingectomy, *PONV* postoperative nausea and vomiting

Data are presented as mean ± standard deviation, median (interquartile range), or number of patients (%) where appropriate

The Study group had significantly less rescued morphine in the first 24 h postoperatively than the Control group [5 (2–9) vs. 8.5 (5–12.8) mg, *P* = 0.013]. The Study group had statistically lower NRS pain scores at 1 h [3 (2–4) vs. 4 (3–5), *P* = 0.007], 2 h [3 (2–4) vs. 3.5 (3–5), *P* = 0.010], 6 h [3 (2–3) vs. 3 (2.3–4), *P* = 0.028], and lower incidence of nausea at 48 h (25.8% vs. 50%, *P* = 0.039) compared to the Control group. No significant differences between groups were observed with regards to pain scores at 24 h and 48 h, the incidence of vomiting, or the need for rescued antiemetics (Table 2).

**Table 2:** Postoperative pain management and adverse events

	Study Group (*n* = 31)	Control Group (*n* = 32)	Median difference (95% CI)	*P* value
Morphine consumption at 0–24 h (mg)	5 (2–9)	8.5 (5–12.8)	-3 (− 6 - − 1)	0.013
Morphine consumption at 24–48 h (mg)	1 (0–1)	0 (0–2)	0 (0–0)	0.913
NRS at 1 h	3 (2–4)	4 (3–5)	-1 (− 2–0)	0.007
NRS at 2 h	3 (2–4)	3.5 (3–5)	-1 (− 2–0)	0.010
NRS at 6 h	3 (2–3)	3 (2.3–4)	-1 (− 1–0)	0.028
NRS at 24 h	2 (1–3)	2 (1.5–3)	0 (− 1–0)	0.355
NRS at 48 h	1 (1–2)	1 (1–2.4)	0 (− 1–0)	0.283
Nausea 0–48 h (n/%)	8 (25.8%)	16 (50.0%)		0.048
Vomiting 0–48 h (n/%)	7 (21.9%)	7 (21.9%)		> 0.999
Rescue antiemetics 0–48 h (n/%)	8 (25.0%)	6 (18.8%)		0.545

Data are presented as mean ± standard deviation or number of patients (%) where appropriate

*NRS* numerical rating scale, *CI* confidence interval

There were no significant differences in the time to first ambulation [20 (18–21) h vs. 19.5 (17–21.8) h, *P* = 0.989], and time to first flatus [26(21–36) h vs. 26(22–37.6) h, *P* = 0.929] between the two groups. The patient satisfaction score was higher in the Study group [10 (10–10) vs. 10 (8–10), *P* = 0.041].

The SF-36 bodily pain score on POD 30 was higher in the Study group (59 ± 13 vs. 49 ± 16, *P* = 0.023). The global QoR-40 score at 24 h and SF-36 scores on POD 30 were comparable between the two groups. (Tables 3 and 4).

**Table 3:** Postoperative QoR-40 scores at 24 h

	Study Group (*n* = 31)	Control Group (*n* = 32)	Mean or Median difference (95% CI)	*P* value
Global QoR-40 score	176.3 ± 15.9	170.4 ± 17.6	5.9 (−2.5–14.4)	0.164
Emotional state	37.4 ± 5.3	35.5 ± 6.5	1.9 (−1.2–4.9)	0.219
Physical comfort	54 (49–56)	51.5 (45.5–55)	2 (− 1–5)	0.207
Psychological support	34 (31–35)	33.5 (31–35)	0 (0 - -2)	0.245
Physical independence	24 (22–25)	24.5 (21.3–25)	0 (−1–1)	0.953
Pain	31 (29–34)	30 (28.3–32)	1 (−1–3)	0.203

Data are presented as mean ± standard deviation or median (interquartile range) where appropriate

*QoR-40* Quality of Recovery 40, *CI* confidence interval

**Table 4:** Postoperative SF-36 scores on POD 30

	Study Group (*n* = 28)	Control Group (*n* = 29)	Mean or Median difference (95% CI)	*P* value
Global SF-36 score	478 ± 112	426 ± 145	51.6 (− 17.3–120.5)	0.139
Physical composite score	218 ± 49	195 ± 66	23.1 (− 7.7–54.0)	0.138
Physical Functioning	71 ± 18	63 ± 21	7.8 (− 2.6 - -18.2)	0.140
Role- Physical	0 (0–25)	0 (0–25)	0 (0–0)	0.561
Bodily Pain	59 ± 13	49 ± 16	9.1 (1.3–17.0)	0.023
General Health	70 ± 16	67 ± 18	3.0 (− 6.2–12.1)	0.517
Mental composite score	260 ± 76	231 ± 89	28.5 (− 15.6–72.5)	0.201
Vitality	68 ± 20	58 ± 22	9.9 (− 1.1–20.9)	0.075
Social Functioning	67 ± 22	57 ± 29	9.6 (− 4.2–23.4)	0.167
Role- Emotional	50.2 (0–100)	33 (0–100)	0 (0–33)	0.737
Mental Health	76 ± 15	70 ± 20	5.8 (− 3.6–15.1)	0.220

Data are presented as mean ± standard deviation or median (interquartile range) where appropriate

*SF-36* short form 36; *POD* postoperative day; *CI* confidence interval

## Discussion

Our study demonstrated additional benefit of preoperative TAP block when used as part MMA after open gynecologic surgery. When TAP block was added to MMA, rescued morphine within the first 24 h was significantly reduced compared to the control group. Also, the incidence of nausea within the first 48 h and pain intensity up to postoperative 30 days were significantly reduced.

TAP block is a peripheral nerve block designed to anesthetize the afferent nerves supplying the anterior abdominal wall from the T6 to L1 thoracolumbar nerve [10]. TAP block provides excellent analgesia to the skin and musculature of the anterior abdominal wall. Thus, TAP block could confer obvious benefit for patients undergoing abdominal hysterectomy with moderate and severe pain [16].

Since MMA with non-opioid medications has become normative in clinical practice, additional effect of TAP block should be further demonstrated. A randomized trial showed the effective analgesia of TAP block with 0.75% ropivacaine in total abdominal hysterectomy. Morphine requirement for up to 48 h was reduced in the TAP block group [17]. In our present study, pre-operative TAP block showed preventive analgesic effect within MMA. Rescued morphine and pain scores within the first 24 h were significantly reduced. This can be explained that a single-shot TAP with 0.375% ropivacaine could produce effective analgesia for only up to 24 h. Of note, in contrast to previous studies that have revealed high dose morphine consumption for open gynecologic surgery [8, 9, 17], our result showed effective analgesia with relatively low dose of morphine in same population. We assume that this finding could be attributed to interindividual variability in pain perception and response to opioid treatment.

We also note some inconsistent results for TAP block in abdominal surgeries. Gasanova et al. [18] demonstrated that surgical site infiltration provided superior pain relief and reduced opioid consumption for up to 48 hours compared to TAP block. They used different local anesthetics for two groups (a long-lasting liposomal formulation of bupivacaine for surgical site infiltration vs. 0.5% bupivacaine for TAP block) which may explain these findings. Griffiths et al. [19] failed to show any additional benefit from a standard posterior approach TAP block in patients receiving MMA for midline laparotomy. No significant difference in morphine consumption at 2 h or 24 h was observed between placebo and TAP block group. They attributed these to obese patients and heterogeneity in surgical procedures.

ERAS programs aim to accelerate and support patient’s return to full functional recovery. Patient-reported outcomes measure any aspect of a patient’s health status. The QoR-40 is a 40-item quality of recovery score measuring five dimensions. It was specifically designed to evaluate a patient’s early postoperative recovery after different type of surgery. A negative association between the global score and duration of hospital day was demonstrated in different types of surgery [20, 21]. The SF-36 is a 36-item health status questionnaire measuring eight dimensions of quality of recovery: physical functioning, role-physical, bodily pain, general health, mental health, role-emotional, social functioning, and vitality. The first four subscales comprise the physical dimension and the latter four comprise the mental dimension. The high score indicates a more favorable health state [22]. Higher pain scores and more complications are correlated with poor quality of recovery in the immediate postoperative period [23, 24]. A poor-quality recovery measured by lower QoR-40 score in the early postoperative period can predict a poor quality of life measured by the SF-36 at 3 months after surgery [25].

De Oliveira GS Jr. et al. [26] demonstrated that preoperative TAP block with 0.5 and 0.25% ropivacaine leads to a better quality of recovery in patients undergoing laparoscopic hysterectomy. Effective analgesia was associated with higher QoR-40 scores in these patients. However, Kane et al. [27] revealed similar QoR-40 scores when utilizing TAP block in laparoscopic hysterectomy patients. The TAP block was accomplished at the end of the procedure, and the QoR-40 score was 168 (125–195) versus 169.5 (116–194) in the TAP block and no-block group respectively.

Our study did not show any differences in QoR-40 score at 24 h and SF-36 score on POD 30 between two groups. However, it is noteworthy that the TAP block group had higher SF-36 bodily pain subscale score compared to the Control group. Nieboer et al. [27] demonstrated that patients who underwent laparoscopic hysterectomy had a better quality of life up to 4 years compared with abdominal hysterectomy. Total SF-36 score was higher in patients after laparoscopic surgery and chronic abdominal pain may contribute to this difference [28]. The SF-36 bodily pain subscale is used to assess a composite score of pain intensity along with interference with daily work. A higher pain score means less impact on daily work and life. The benefit of TAP block on chronic postsurgical pain in this population need to be demonstrated in future studies.

This study has several limitations. First, TAP block was accomplished after the induction of anesthesia. Hence, we did not assess the definite blockade range before surgery. Secondly, only one dosage of ropivacaine was used in this trial, thus further investigations are needed to demonstrate optimal concentration of local anesthetic in TAP block. Finally, the QoR-40 and SF-36 score were secondary outcomes and the power was insufficient to detect a difference between the two groups. Future research focused on better quality of recovery provided by multimodal analgesia for this population may be desirable.

## Conclusion

Preoperative TAP block had additional analgesic effect for open gynecological surgery when used as part of multimodal analgesia. Rescued morphine within 24 h was significantly reduced and the SF-36 bodily pain dimension at 30 days after surgery was significantly improved.

## Data Availability

The datasets that support the finding of the current study are available from the corresponding author on reasonable request.

## Abbreviations

ERAS: Enhanced recovery after surgery
TAP: Transversus abdominis plane
MMA: Multimodal analgesia
QoR-40: 40-item quality of recovery score
SF-36: Short-form health survey
PCA: Patient-controlled analgesia
PACU: Post-anesthesia care unit
POD: Postoperative day
NRS: Numerical rating scale
